# An RB1CC1 Missense Variant in Nova Scotia Duck Tolling Retrievers with Degenerative Encephalopathy

**DOI:** 10.3390/genes16030269

**Published:** 2025-02-25

**Authors:** Juyuan Guo, Garrett Bullock, Dennis P. O’Brien, Gary S. Johnson, Martin L. Katz

**Affiliations:** 1 Canine Genetics Laboratory, Department of Veterinary Pathobiology, College of Veterinary Medicine, University of Missouri, Columbia, MO 65211, USA; guoj@missouri.edu (J.G.); gebkd2@missouri.edu (G.B.); 2 Department of Veterinary Medicine and Surgery, College of Veterinary Medicine, University of Missouri, Columbia, MO 65211, USA; obriend@missouri.edu (D.P.O.);; 3 Neurodegenerative Diseases Research Laboratory, Department of Ophthalmology, University of Missouri, Columbia, MO 65212, USA

**Keywords:** autophagy, neurodegeneration, canine, whole genome sequencing, lipofuscin

## Abstract

Background/Objectives: A slowly progressive hereditary neurological disorder classified as degenerative encephalopathy (DE) occurs in Nova Scotia Duck Tolling Retrievers. The disease is characterized by frequent episodes of pronounced involuntary movements during sleep, cognitive impairment, anxiety, heightened sensitivity to sensory stimuli, and compulsive behaviors. The clinical signs are accompanied by the degeneration of several brain regions. A study was undertaken to identify the molecular genetic basis of this disorder. Methods: Whole genome sequences (WGSs) from the DNA of affected and unaffected Nova Scotia Duck Tolling Retrievers were aligned to the Dog10K_Boxer_Tasha reference genome assembly and to the WGSs of 334 additional control dogs generated by this laboratory. Results: A missense C>T variant was identified in *RB1CC1* exon 22 chromosome 29:4891014 that was uniquely homozygous in the affected dog. This variant predicts a p.G1503R change in the amino acid sequence of RB1CC1. Genotyping of 2950 Nova Scotia Duck Tolling Retrievers at the variant locus found complete concordance between the disease phenotype and *RB1CC1* genotype. Conclusions: RBCC1 is an essential component of a protein complex that mediates the initiation of autophagosome formation. Therefore, it appears likely that the disease results, at least in part, from impaired autophagy. Consistent with this possibility, brain neurons of an affected dog were found to contain abnormal lysosomal storage body-like inclusions. This disorder could serve as a valuable model to elucidate the mechanisms underlying human diseases associated with impaired autophagy. Identification of the disease-causing DNA sequence variant will enable owners of Nova Scotia Duck Tolling Retrievers to screen their dogs for the *RB1CC1* risk variant.

## 1. Introduction

In 2016, a slowly progressive neurological disorder classified as degenerative encephalopathy was described in Nova Scotia Duck Tolling Retrievers [1]. The most consistent sign is frequent episodes of pronounced involuntary movements during sleep. Affected dogs also exhibit cognitive impairment, anxiety, heightened sensitivity to sensory stimuli, and compulsive behaviors. As the disease progresses, some affected dogs exhibit aggressive behavior, gait abnormalities, and urinary and fecal incontinence. The severity of signs increases over time. Magnetic resonance imaging of the brains of affected dogs revealed pronounced abnormalities of the caudate nucleus as well as other more subtle abnormalities elsewhere in the brain. Due to the progression of the disease signs, some of the affected dogs were humanely euthanized. Postmortem examination of the brains revealed cavitation lesions in the caudate nucleus. Based on the pattern of inheritance, the disorder appears to be an autosomal recessive trait. Studies were undertaken to identify the molecular genetic basis of this disorder.

## 2. Materials and Methods

### 2.1. Identification of Cases and Controls

The hallmark of Nova Scotia Duck Tolling Retriever degenerative encephalopathy (DE) is pronounced involuntary movements during sleep [1]. The onset of signs has been reported to occur as early as 2 months of age and as late as 5 years of age [1]. Therefore, for this study, cases included dogs that had exhibited the marked and progressive sleep disturbance sign characteristic of this disorder by 5 years of age. Control Nova Scotia Duck Tolling Retrievers consisted of dogs that had not exhibited signs of the disorder by 6 years of age. DNA samples from a total of 2950 purebred Duck Tolling Retrievers were utilized for this study. Disease status was assessed with a questionnaire completed by the dogs’ owners in which they indicated whether the dogs exhibited signs characteristic of the DE disorder. In addition, DNA samples from 334 dogs from other breeds that did not exhibit signs of DE were utilized for whole genome sequence analyses. Owners of all dogs utilized in this study provided written informed consent for the use of the samples and health information in any studies to be conducted by the laboratory. Genotype information linked to specific dogs was provided only to those dogs’ owners.

### 2.2. Molecular Genetic Analyses

DNA from affected and unaffected Nova Scotia Duck Tolling Retrievers and unaffected dogs of other breeds was isolated from EDTA anticoagulated blood or FTA Elute cards [2,3], collected with the informed consent of the owners. Whole genome sequences were generated from DNA samples from selected dogs by the University of Missouri Genomics Technology Core Facility from Illumina TruSeq PCR-free paired-end libraries. The sequence reads were mapped to canine reference genome assembly Dog10K_Boxer_Tasha with the Burrows-Wheeler Aligner and sorted with SAMtools (ver. 1.11). PCR duplicates were marked with Picard tools (ver. 2.23.8). Realignment, recalibration, and variant calling were performed with the Genome Analysis Tool Kit (GATK ver. 3.8) pipeline. An additional 334 canine whole genome sequences previously generated by the University of Missouri Canine Genetics Laboratory were used as additional unaffected controls to help identify variants that were common in the general canine population. All whole genome sequences utilized in this study have been deposited in the NCBI Sequence Read Archive (SRA) (https://trace.ncbi.nlm.nih.gov/Traces/sra/sra.cgi; accessed 19 February 2025) (Supplementary Table S1). GATK HaplotypeCaller in the gVCF mode was utilized to identify variants in the affected dog relative to the reference cohort. GATK CombineGVCFs was used to join the sample gVCF files which were then jointly genotyped with GATK GenotypeGVCFs. Predicted effects of the called variants on the functions of the encoded proteins were determined using SnpEff software [4] in conjunction with Ensembl annotation. Low-quality variants were removed and annotated variants were extracted for the affected sample with SnpSift software [4]. The annotated output was exported to a Microsoft Excel spreadsheet with GATK VariantsToTable. The Integrative Genomics Viewer (IGV, ver. 2.8.10) was used to validate candidate variants.

A homozygous missense variant in *RB1CC1* exon 22 chromosome 29:4891014, C>T (UU_Gfam_GSD_1.0/CanFam4 coordinate 29:4747722; Ensembl Gene ID: ENSCAFG00805024257) was identified in the whole genome sequence of an affected dog (see Section 3). This variant was not present in the control dog whole genome sequences. To verify the candidate *RB1CC1* variant, Sanger sequencing of the region of *RB1CC1* surrounding position 29:4891014 was performed on DNA samples from affected dogs using PCR primers: 5′-TATATGGCTGAACATTGCAGA-3′ and 5′-TCCGAATGCAGAAAATACAAGG-3′. The PCR amplifications were conducted in 30 µL volumes with a DNA Polymerase Kit (Promega, Madison, WI, USA) and included an initial denaturation at 95 °C for 2 min, followed by 40 cycles of denaturation at 95 °C for 15 s, primer annealing at 60 °C for 15 s, extension at 72 °C for 30 s, and a final extension at 72 °C for 2 min.

An allelic discrimination assay was designed for genotyping individual dogs for the *RB1CC1* variant. The PCR primer sequences were 5′-GGGTAGCAGTAAGCCTTTAGGATAATAAAT-3′ and 5′-CGCTCGTCTAGGATGATGAGTAC-3′. The competing probe sequences were 5′-VIC-TTCAGGTGGGAGATTT-NFQ-3′ (reference allele) and 5′-FAM-TTTCAGGTGAGAGATTT-NFQ-3′ (variant allele). These amplifications were conducted in 20 µL volumes with a TaqMan Genotyping Master Mix (Applied Biosystems, Waltham, MA, USA) and included an initial denaturation at 95 °C for 10 min, followed by 40 cycles of denaturation at 95 °C for 15 s, primer annealing at 60 °C for 1 min, and a final extension at 60 °C for 30 s on a StepOnePlus Real-Time PCR system (Applied Biosystems).

### 2.3. Electron Microscopy

Cavitation and astrocytosis of the caudate nucleus are the most pronounced brain lesions in Nova Scotia Duck Tolling Retrievers suffering from degenerative encephalopathy [1]. To determine whether this pathology was accompanied by ultrastructural abnormalities in caudate nucleus neurons, brain tissue was collected from an affected dog that was humanely euthanized due to the progression of disease signs. The caudate nucleus was fixed in 2% glutaraldehyde, 1.2% paraformaldehyde, 120 mM sodium cacodylate, 1 mM CaCl_2_, pH 7.4 for 7 days. The sample was then rinsed in 170 mM sodium cacodylate, post-fixed with 1% osmium tetroxide, and embedded in Embed 812 resin (Electron Microscopy Sciences cat. no. 14120, 14900 (Hatfield, PA, USA). Sections of the sample were cut at thicknesses of 70 to 90 nm and stained with uranyl acetate and lead citrate. The sections were examined with a JEOL JEM-1400 transmission electron microscope equipped with a Gatan digital camera.

## 3. Results

**Figure 1:**
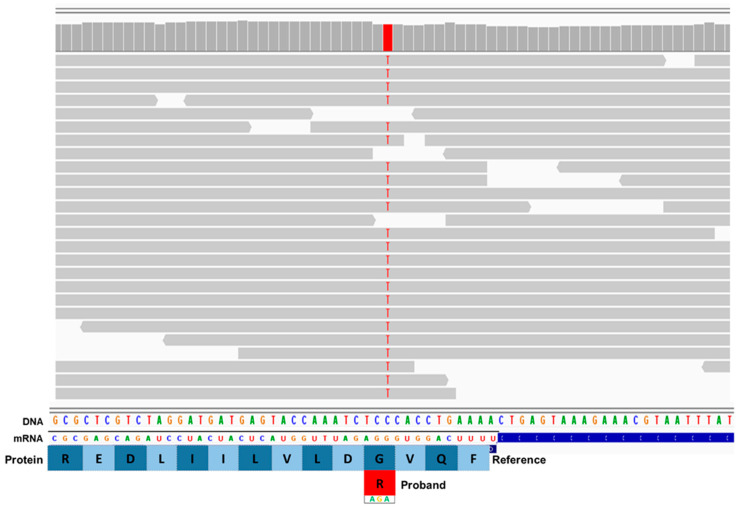
Screenshot of the proband’s whole genome sequence reads aligned to the reference sequence in the vicinity of position on 4,891,014 chromosome 22 (red bar at top), as viewed with the Integrative Genomics Viewer. The variant T is highlighted in red. The reference DNA and mRNA sequences are shown, along with the predicted protein amino acid sequence. The codon change from GGA to AGA predicts an amino acid change from glycine (G) to arginine (R).

**Figure 2:**
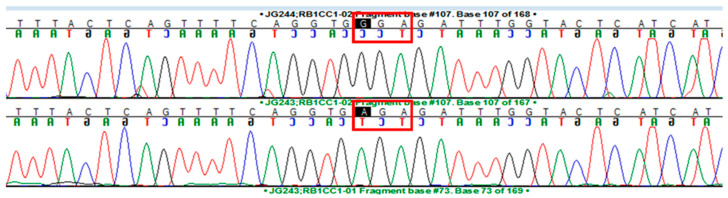
Automated Sanger sequence electrophoretograms of DNA from an unaffected dog (top line) and proband (bottom line). The codon for RB1CC1 p.1503 is GGA in the unaffected dog and AGA in the affected dog (red boxes), confirming the finding from the whole genome sequence analysis.

Whole genome sequences of the affected and unaffected dogs were generated with target 30-fold average coverage. The sequence of the affected dog contained a C-to-T transition at position 4,891,014 on chromosome 22 that was uniquely homozygous in the proband relative to the 335 reference whole genome sequences. The variant is predicted to produce a p.G1503R change in the RB1 inducible coiled-coil protein 1 (RB1CC1). The validity of this variant call was confirmed through an inspection of aligned reads from the affected dog’s whole genome sequence to the Tasha reference sequence from positions 4,890,982 to 4,891,047 on chromosome 22 using the Integrative Genomics Viewer (Figure 1), and via Sanger sequencing (Figure 2).

To assess the concordance between the *RB1CC1* 29:4891014 genotype and phenotype, a total of 2950 Nova Scotia Duck Tolling Retrievers were genotyped with the allelic discrimination assay that distinguished between the reference and variant alleles. Of these dogs, 2590 were homozygous for the reference C allele, 336 were heterozygous, and 24 were homozygous for the T risk allele. None of the dogs homozygous for the reference allele or that were heterozygous exhibited disease signs, including the parents of affected dogs. The only potential exception to concordance between genotype and phenotype was one dog that exhibited signs similar to those of dogs with degenerative encephalopathy but was homozygous for the reference allele. Although this dog did exhibit running movements during sleep, it also exhibited sudden-onset bouts of aggression and altered behavior that are not typical of the disorder. Due to these phenotypic differences, this dog was not considered to suffer from DE. All of the unaffected dogs of other breeds in the reference cohort were homozygous for the reference C allele.

The PolyPhen-2 tool (PolyPhen-2: prediction of functional effects of human nsSNPs (harvard.edu; accessed 10 December 2024) was employed to estimate the functional effect of the *RB1CC1* variant on the encoded protein. The PolyPhen-2 score was 0.9999, which predicted that the effect of the variant was very likely to be deleterious.

The amino acid sequence predicted from the proband WGS from p.1470 to p.1503 was aligned to the predicted corresponding *RB1CC1* amino acid sequences of 50 mammalian species in the NCBI database. The predicted amino acid sequences were completely conserved across all 50 species except that all but the proband had glycine at the position corresponding to canine p.1503 rather than arginine predicted from the proband sequence.

**Figure 3:**
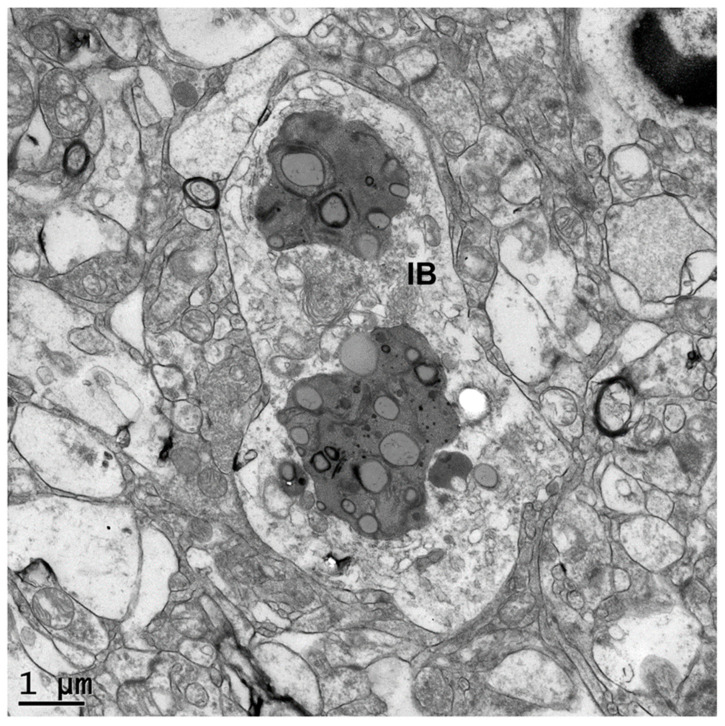
Electron micrograph of a disease-specific inclusion body (IB) in a caudate nucleus neuron of the proband. The contents of the inclusion body are quite heterogeneous in appearance.

**Figure 4:**
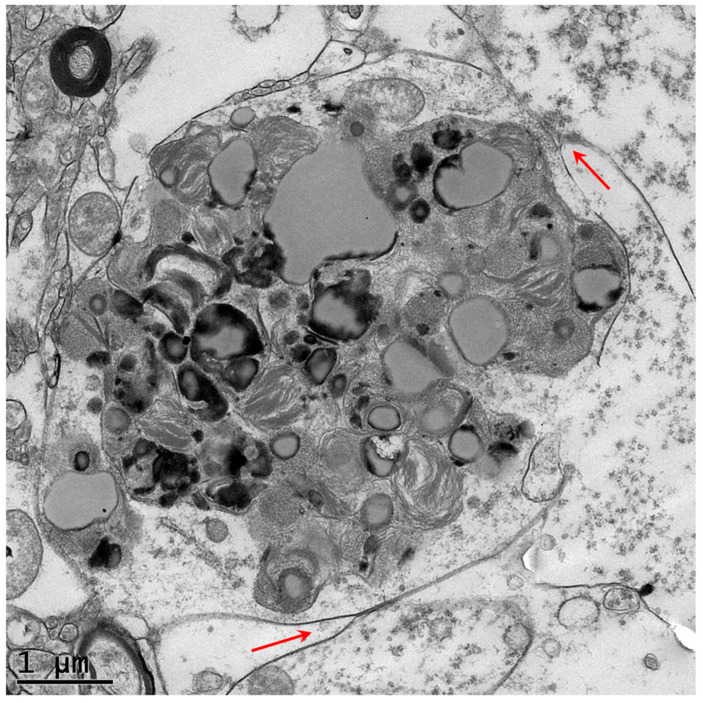
Electron micrograph of a disease-specific inclusion body in a caudate nucleus neuron of the proband. Finger-like membrane-bounded cytoplasmic extensions partially engulf the inclusion body (red arrows).

**Figure 5:**
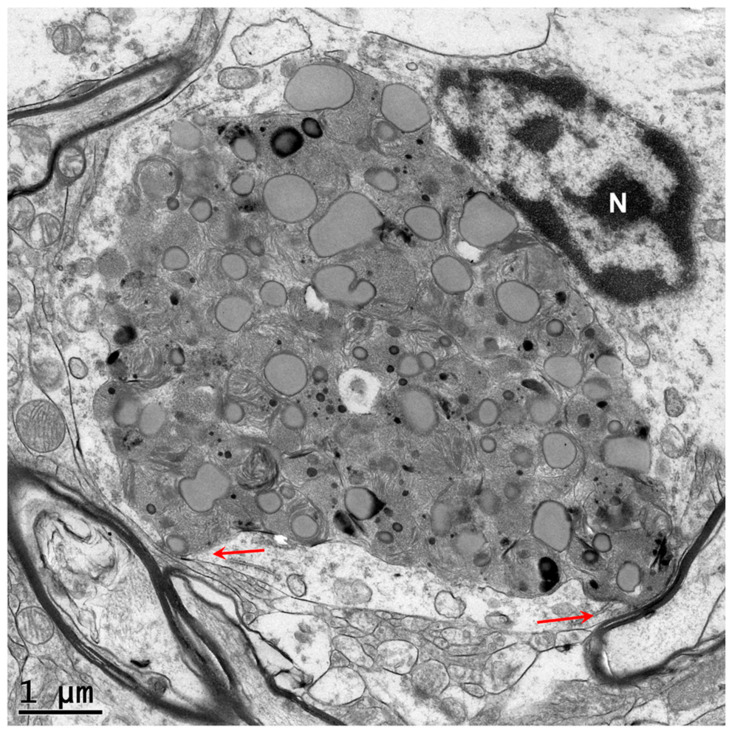
Electron micrograph of a disease-specific inclusion body in a caudate nucleus neuron of the proband. A membrane-bounded structure abuts the inclusion body (red arrows).

Neurons of the proband’s caudate nucleus contained large membrane-bounded inclusions with heterogeneous contents similar to those that accumulate in some lysosomal storage disorders [5,6,7] (Figure 3, Figure 4 and Figure 5). Within these inclusion bodies were membrane-like components, lipid droplets, amorphous granular materials, and very electron dense matter. In some planes of the section, some of the large inclusion bodies were at least partially surrounded by membrane-bounded finger-like cytoplasmic extensions (Figure 4 and Figure 5).

## 4. Discussion

**Figure 6:**
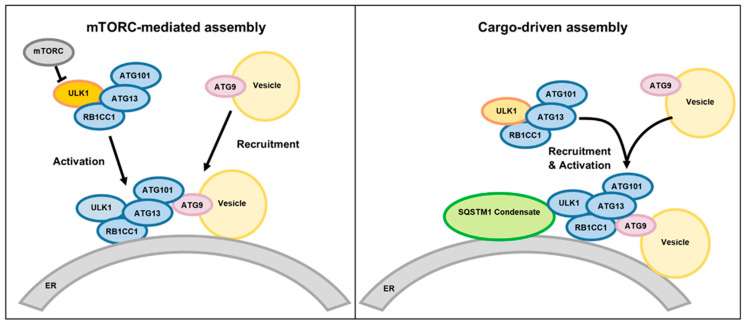
Alternative mechanisms of macroautophagy initiation. In mTORC-mediated initiation of autophagosome formation, intracellular signaling by factors such as starvation inhibit the mTORC-mediated phosphorylation of ULK1, resulting in the dephosphorylation of ULK1, which activates the ULK complex which then binds to the endoplasmic reticulum (ER) and recruits ATG9-containing membrane vesicles. In a cargo-driven process, the SQSTM1 condensate, consisting of a complex of specific proteins and ubiquitinated cargo molecules, binds to the ER and recruits the ULK complex and ATG-containing vesicles to initiate autophagosome formation. (Adapted from [13]).

The RB1-inducible coiled-coil 1 protein (RB1CC1; also known as FIP200) plays a central role in macroautophagy, a process by which intracellular components are engulfed into membrane-bounded organelles (autophagosomes) and degraded after fusion of the autophagosomes with lysosomes [8,9,10,11,12,13]. The degradation of damaged cellular components via autophagy is necessary for maintaining cellular homeostasis. The autophagic process is initiated by formation of the ULK protein complex consisting of RB1CC1, ULK1, ATG13, and ATG101 proteins near the endoplasmic reticulum (Figure 6) [8,9,10,11,12,13]. The activation of the ULK complex by intracellular signals starts the process of autophagosome formation. Formation of the double-walled autophagosome membrane is initiated when the activated ULK complex recruits ATG9 protein-containing vesicles to the surface of the endoplasmic reticulum. Many other proteins are involved in subsequent steps of autophagosome formation and function [13].

Consistent with the conclusion that the canine disorder is the result of the *RB1CC1* 29:4891014 C>T variant, variants in autophagy genes have been associated with a number of human autosomal recessive hereditary neurodegenerative diseases. Among these are ataxia with developmental delay [14], mild to severe intellectual disability with ataxia and tremor [15], global developmental delay, seizures, and spastic quadriplegia [16], and spinocerebellar ataxia [17]. Cases of schizophrenia with abnormalities in the dentate gyrus have also been associated specifically with *RB1CC1* variants [18,19]. The importance of RB1CC1-mediated functions in maintaining normal neurological structure and function is supported by a number of gene-knockout and knock-in studies in mice [20,21,22,23,24,25]. For example, the neural-specific deletion of *RB1CC1* resulted in cerebellar atrophy associated with axonal degeneration and neuronal death [25]. These data and the presence of storage body accumulations in neurons of dogs with the RB1CC1 p.G1503R variant support the conclusion that the neurological disorder in these dogs is the result of impaired autophagy. Variants in genes encoding other proteins involved in the autophagy pathway also result in neurological disorders [26,27].

Autophagosomes are double-membraned vesicles. Defects in the later stages of autophagy result in the intracellular accumulation of these organelles. For example, mutations in *TECPR2* that encodes a protein that mediates the fusion of autophagosomes with lysosomes result in the accumulation of double-membrane vesicles [27,28,29]. In contrast, the *RB1CC1* variant was associated with an accumulation of inclusions surrounded by a single membrane, consistent with impairment in the formation of the normal double-membrane structures. The impaired ability to synthesize autophagosomes (and their precursor phagophores) apparently results in cellular components that would normally be degraded by autophagy accumulating in single-membrane bound lipofuscin-like inclusions. Numerous studies indicate that impaired autophagy is associated with the accumulation of lipofuscin-like intracellular inclusions, similar to that observed in neurons of the affected dog evaluated in this study [30,31,32,33,34,35,36,37]. For example, the treatment of retinal pigment epithelial cells in vitro with an inhibitor of autophagy resulted in the increased accumulation of lipofuscin-like inclusions [30]. In addition, mice that were homozygous for a null mutation in *Per1* (Period 1) exhibited impaired autophagy and a massive accumulation of lipofuscin-like material in the hippocampus [35]. The mechanisms by which autophagy defects result in the accumulation of lipofuscin-like inclusions have not been elucidated. It appears that when damaged intracellular components cannot traverse the autophagosome to autolysosome degradative pathway, they are sequestered in inclusion bodies such as those observed in the caudate nucleus of a dog that was homozygous for the *RB1CC1* risk variant. The ultrastructural findings in this study suggest that normal age-related accumulation of lipofuscin may be the result, at least in part, of the insufficient degradation of cellular components via autophagy [34,35,38,39,40].

The change in RB1CC1 (FIP200) amino acid residue at position 1503 from glycine to arginine likely hinders autophagosome formation by altering the interaction of the protein with other components of the initiation complex. A component of this complex is a condensate of a specific group of proteins with ubiquitinated cargo molecules (SQSTM1 condensate) (Figure 5). It was found that the region of RB1CC1 surrounding residue 1503 is involved in the interaction between RB1CC1 and the SQSTM1 protein complex that initiates cargo-driven autophagosome synthesis [41,42,43]. The replacement of glycine with arginine within this region would be expected to alter this interaction, as well as interactions with other components of the autophagosome initiation complex [44].

Although the accumulation of membrane-bound inclusion bodies in neurons of the proband suggests that the autophagy process was impaired, RB1CC1 has also been found to have other functions in addition to its role in autophagy [45,46,47,48,49,50,51,52,53,54,55,56,57,58,59,60]. For example, RB1CC1 has been shown to have autophagy-independent roles in cell adhesion and motility [45], modulating gene expression [48], and immune signaling [52,53]. It is possible that the impairment of these functions by the alteration in the RB1CC1 amino acid sequence contributed to the disease pathology in the affected dogs.

A number of studies have demonstrated the importance of RB1CC1 in many tissues in outside of the central nervous system [20,23,25,61,62,63,64,65,66,67,68,69,70,71,72,73]. For example, variants in *RB1CC1* have been associated with differences in muscle strength in human subjects [74], and conditional *rb1cc1* knockout in mice results in myopathy with the accumulation of inclusion bodies in muscle fibers [70]. Considering the key role that RB1CC1 plays in autophagy and other cellular functions, it is not surprising that multiple tissues are impacted by alterations in the function of this protein. It is possible that some of the signs exhibited by the dogs with the *RB1CC1* missense variant were secondary to pathology outside of the central nervous system. For example, extra-neuronal pathology may have played a role in the gait abnormalities and urinary and fecal incontinence that characterized the later stages of the canine disorder. It will be of interest to evaluate additional tissues from affected dogs, particularly skeletal muscle, should they become available.

Based on our findings, owners of Nova Scotia Duck Tolling Retrievers can now screen their dogs for the *RB1CC1* degenerative encephalopathy risk variant and avoid breeding dogs that could produce affected offspring.

## Data Availability

Whole genome sequence data generated in this study were deposited in the NCBI Sequence Read Archive (see Table S1).

## Supplementary Materials

The following supporting information can be downloaded at: https://www.mdpi.com/article/10.3390/genes16030269/s1, Table S1: NCBI Sequence Read Archive BioSample IDs.
